# Supplementary material for: Bright daytime light enhances circadian amplitude in a diurnal mammal
Source: Proc Natl Acad Sci U S A. 2021 May 24;118(22):e2100094118. doi: 10.1073/pnas.2100094118 (PMC8179182; doi:10.1073/pnas.2100094118)
Supplement: Supplementary File [file pnas.2100094118.sapp.pdf]

PNAS
www.pnas.org

Supplementary Information for

Bright daytime light enhances circadian amplitude in a diurnal mammal

Beatriz Bano-Otalora, Franck Martial, Court Harding, David A. Bechtold, Annette E. Allen, Timothy M. Brown, Mino D. C. Belle, Robert J. Lucas*

*Corresponding author: Robert J. Lucas

Email: robert.lucas@manchester.ac.uk

This PDF file includes:

Figure S1
Table S1

Supplementary Information

Supplementary Figure S1

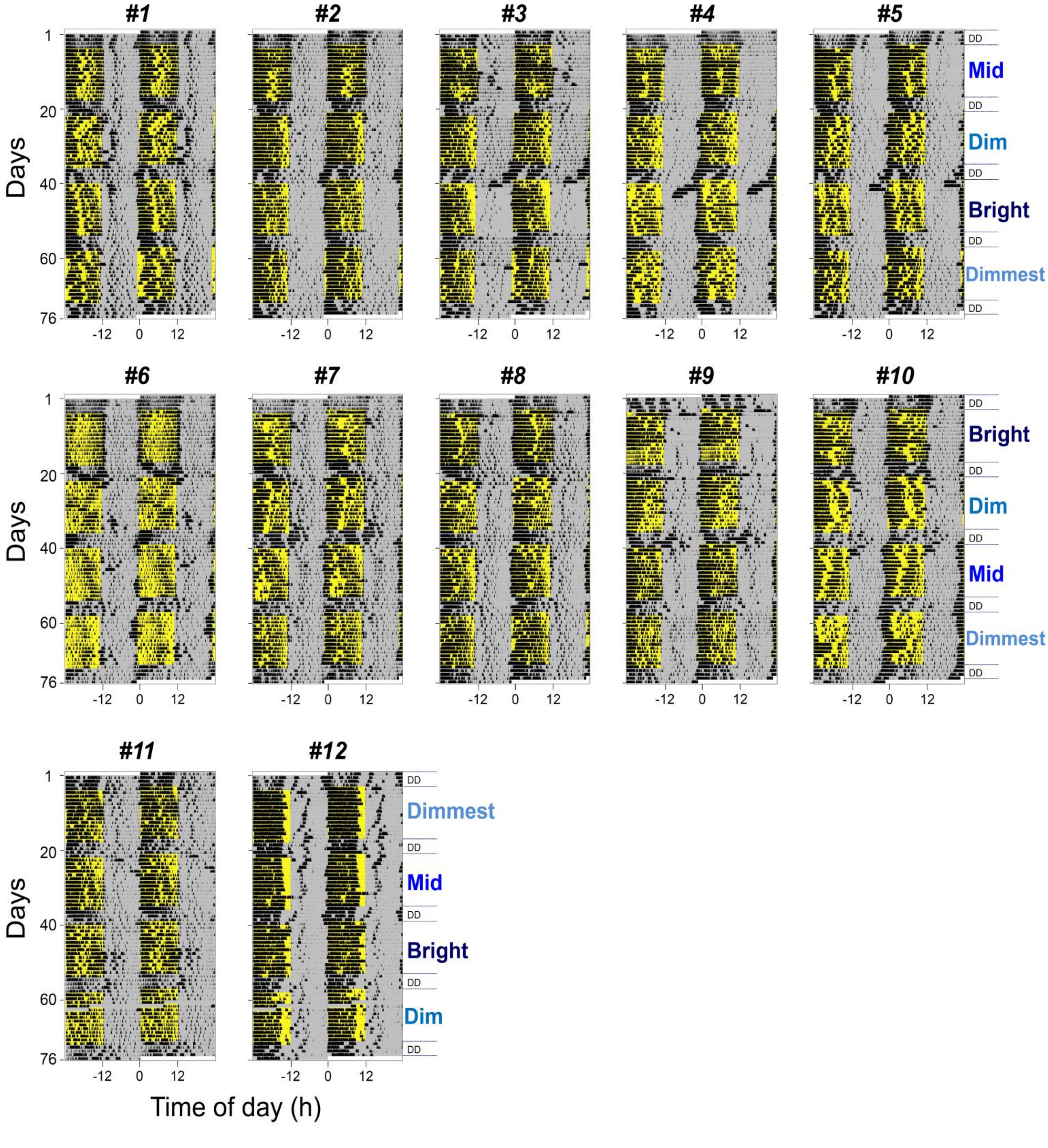

Fig. S1. Impact of increasing daytime light intensity on activity rhythms in *Rhabdomys pumilio*. Double-plotted actograms of general activity for a total of 12 animals under different lighting conditions, over a period of 2.5 months. Time of light exposure is indicated in yellow, and intensity of the light is shown on the left. Entrainment to 12:12 LD cycle at each irradiance ran for 2 weeks followed by 4 days in constant darkness (DD).

Supplementary Table S1

Table S1. Behavioral and physiological rhythms under different daytime irradiances.

n=12	Log Mel Effective photon flux/cm^2/s	Dimmest 12.77	Dim 12.95	Mid 13.66	Bright 14.80	Is slope significantly non-zero?
General Activity	Mean Activity-Light	390.40 ± 26.50	343.70 ± 16.08	392.50 ± 26.01	368.10 ± 20.39	0.997
	Mean Activity-Dark	68.53 ± 7.14	69.21 ± 9.42	60.98 ± 7.26	73.73 ± 11.29	0.708
	Mean-24h	229.50 ± 11.99	206.50 ± 8.68	226.80 ± 12.05	220.90 ± 11.21	0.885
	% Diurnality	84.130 ± 2.614	83.300 ± 2.039	85.740 ± 2.202	83.350 ± 2.501	0.934
	Day-to day Reproducibility	0.668 ± 0.037	0.638 ± 0.042	0.739 ± 0.031	0.718 ± 0.030	0.128
	Intradaily-Variability	0.512 ± 0.057	0.542 ± 0.057	0.439 ± 0.057	0.446 ± 0.051	0.219
Wheel Running Activity	Mean Activity-Light	684.90 ± 71.64	598.60 ± 69.28	542.70 ± 77.12	625.60 ± 107.20	0.768
	Mean Activity-Dark	39.13 ± 12.58	30.54 ± 12.31	33.94 ± 9.20	60.13 ± 13.97	0.113
	Mean-24h	362.00 ± 36.60	314.50 ± 35.83	288.30 ± 39.67	342.90 ± 53.33	0.952
	Total Activity-Light	16438.0 ± 1719.0	14365.0 ± 1663.0	13025.0 ± 1851.0	15015.0 ± 2573.0	0.768
	Total Activity-Dark	939.2 ± 302.0	732.9 ± 295.4	814.6 ± 220.7	1443.0 ± 335.2	0.113
	Total Activity -24h	17377.0 ± 1757.0	15098.0 ± 1720.0	13840.0 ± 1904.0	16458.0 ± 2560.0	0.952
	% Diurnality	91.930 ± 3.675	94.920 ± 1.820	90.950 ± 4.215	87.130 ± 4.366	0.175
	Day-to day Reproducibility	0.648 ± 0.039	0.621 ± 0.058	0.628 ± 0.036	0.646 ± 0.029	0.867
	Intradaily-Variability	0.512 ± 0.103	0.466 ± 0.059	0.498 ± 0.059	0.534 ± 0.077	0.661
Body Temperature	Mean-Light	37.98 ± 0.11	37.92 ± 0.09	38.12 ± 0.10	38.16 ± 0.11	0.090
	Mean-Dark	35.62 ± 0.09	35.64 ± 0.11	35.57 ± 0.07	35.59 ± 0.08	0.725
	Mean-24h	36.80 ± 0.08	36.78 ± 0.09	36.85 ± 0.07	36.88 ± 0.08	0.364
	Day-to day Reproducibility	0.782 ± 0.025	0.781 ± 0.026	0.840 ± 0.016	0.844 ± 0.010	***0.012***
	Intradaily-Variability	0.340 ± 0.027	0.346 ± 0.026	0.275 ± 0.024	0.259 ± 0.017	***0.005***
Sustained Immobility	Mean-Light	0.264 ± 0.029	0.265 ± 0.018	0.212 ± 0.027	0.200 ± 0.016	***0.020***
	Mean-Dark	0.768 ± 0.012	0.751 ± 0.022	0.771 ± 0.021	0.768 ± 0.027	0.768
	Mean-24h	0.516 ± 0.011	0.508 ± 0.015	0.491 ± 0.016	0.484 ± 0.016	0.098
	Day-to day Reproducibility	0.639 ± 0.035	0.620 ± 0.031	0.743 ± 0.029	0.745 ± 0.022	***0.002***
	Intradaily-Variability	0.657 ± 0.056	0.692 ± 0.057	0.506 ± 0.061	0.508 ± 0.052	***0.017***

Data are expressed as mean ± SEM (n=12); Significant p values ($p<0.05$) are in bold.
